# Prognostic impact of preoperative atrial fibrillation in patients undergoing heart surgery in cardiogenic shock

**DOI:** 10.1038/s41598-023-47642-3

**Published:** 2023-12-09

**Authors:** Mariusz Kowalewski, Giuseppe M. Raffa, Michał Pasierski, Michalina Kołodziejczak, Radosław Litwinowicz, Wojciech Wańha, Wojciech Wojakowski, Jan Rogowski, Marek Jasiński, Kazimierz Widenka, Tomasz Hirnle, Marek Deja, Krzysztof Bartus, Roberto Lorusso, Zdzisław Tobota, Bohdan Maruszewski, Piotr Suwalski, KROK Investigators, Lech Anisimowicz, Andrzej Biederman, Dariusz Borkowski, Mirosław Brykczyński, Paweł Bugajski, Marian Burysz, Paweł Cholewiński, Romuald Cichoń, Marek Cisowski, Antoni Dziatkowiak, Tadeusz Gburek, Witold Gerber, Leszek Gryszko, Ireneusz Haponiuk, Piotr Hendzel, Stanisław Jabłonka, Krzysztof Jarmoszewicz, Ryszard Jaszewski, Marek Jemielity, Ryszard Kalawski, Bogusław Kapelak, Maciej A. Karolczak, Jacek Kaperczak, Piotr Knapik, Michał Krejca, Wojciech Kustrzycki, Mariusz Kuśmierczyk, Paweł Kwinecki, Leszek Markuszewski, Maurycy Missima, Jacek J. Moll, Wojciech Ogorzeja, Jacek Pająk, Michał Pasierski, Wojciech Pawliszak, Edward Pietrzyk, Grzegorz Religa, Jacek Różański, Jerzy Sadowski, Girish Sharma, Janusz Skalski, Jacek Skiba, Ryszard Stanisławski, Janusz Stążka, Sebastian Stec, Piotr Stępiński, Grzegorz Suwalski, Kazimierz Suwalski, Łukasz Tułecki, Waldemar Wierzba, Michał Wojtalik, Stanisław Woś, Michał Oskar Zembala, Piotr Żelazny

**Affiliations:** 1 Clinical Department of Cardiac Surgery and Transplantology, Centre of Postgraduate Medical Education, National Medical Institute of the Ministry of Interior and Administration, Wołoska 137 Str, 02-507 Warsaw, PL Poland; 2 Cardio-Thoracic Surgery Department, Heart and Vascular Centre, Maastricht University Medical Centre, Maastricht, The Netherlands; 3 Department for the Treatment and Study of Cardiothoracic Diseases and Cardiothoracic Transplantation, IRCCS-ISMETT, Palermo, Italy; 4 Department of Anaesthesiology and Intensive Care, Ludwik Rydygier Collegium Medicum, Nicolaus Copernicus University, Antoni Jurasz University Hospital No. 1, Bydgoszcz, Poland; 5 Department of Cardiac Surgery, Regional Specialist Hospital, Grudziądz, Poland; 6 Department of Cardiology and Structural Heart Diseases, Medical University of Silesia, Katowice, Poland; 7 Department of Cardiac and Vascular Surgery, Medical University of Gdansk, Gdansk, Poland; 8 Department and Clinic of Cardiac Surgery, Wroclaw Medical University, Wroclaw, Poland; 9 Clinical Department of Cardiac Surgery, District Hospital No. 2, University of Rzeszów, Rzeszów, Poland; 10 Department of Cardiosurgery, Medical University of Bialystok, Bialystok, Poland; 11 Department of Cardiac Surgery, Upper-Silesian Heart Center, Katowice, Poland; 12 Department of Pediatric Cardiothoracic Surgery, The Children’s Memorial Health Institute, Warsaw, Poland; 13 Thoracic Research Centre, Collegium Medicum Nicolaus Copernicus University, Innovative Medical Forum, Bydgoszcz, Poland; 14 Department of Cardiac Surgery, University Hospital, Bydgoszcz, Poland; 15 Cardiac Surgery Department, Medicover Hospital, Warsaw, Poland; 16 Department of Cardiac Surgery, Masovian Specialistic Hospital of Radom, Radom, Poland; 17 Department of Cardiac Surgery, Pomeranian Medical University, Szczecin, Poland; 18 Department of Cardiosurgery, J. Struś Hospital, Poznań, Poland; 19 Department of Cardiac Surgery, Medinet Heart Center Ltd, Wroclaw, Poland; 20 Department of Cardiovascular Surgery, University Clinical Center of the Medical University of Warsaw, Warsaw, Poland; 21 Department of Cardiac Surgery, American Heart of Poland, Bielsko-Biała, Poland; 22 Department of Cardiovascular Surgery and Transplantology, Jagiellonian University Medical College, John Paul II Hospital, Krakow, Poland; 23 Department of Cardiac Surgery, The Pope John Paul II Province Hospital, Zamość, Poland; 24 Department of Cardiac Surgery, Military Institute of Medicine, Warsaw, Poland; 25 Department of Pediatric Cardiac Surgery, Pomeranian Traumatology Center, Gdańsk, Poland; 26 Medical University of Warsaw, Warsaw, Poland; 27 Cardiac Surgery Department, Medical University of Lublin, Lublin, Poland; 28 Department of Cardiac Surgery, Ceynowa Specialist Hospital in Wejherowo, Wejherowo, Poland; 29 Lower Silesian Center for Heart Diseases, Nowa Sól, Poland; 30 Department of Cardiac Surgery, Medical University of Lodz, Lodz, Poland; 31 Department of Cardiac Surgery and Transplantology, Poznan University of Medical Sciences, Poznań, Poland; 32 Department of Cardiac and General Pediatric Surgery, Medical University of Warsaw, Warsaw, Poland; 33 Department of Cardiac Surgery, Institute of Medical Sciences, University Hospital, University of Opole, Opole, Poland; 34 Department of Anesthesiology and Intensive Therapy, Silesian Centre for Heart Diseases in Zabrze, Medical University of Silesia, Katowice, Poland; 35 Department of Cardiac Surgery, Wroclaw Medical University, Wroclaw, Poland; 36 Department of Cardiac Surgery and Transplantology, National Institute of Cardiology, Warsaw, Poland; 37 Department of Medicine, Faculty of Medical Sciences and Health Sciences, Kazimierz Pulaski University of Technology and Humanities in Radom, Radom, Poland; 38 Cardiology and Cardiac Surgery Department, 11th Military Research Hospital and Polyclinic IPHC in Bydgoszcz, Bydgoszcz, Poland; 39 Department of Cardiac Surgery, Polish Mother’s Memorial Hospital Research Institute, Lodz, Poland; 40 Department of Cardiac Surgery, Swietokrzyskie Cardiology Center, Kielce, Poland; 41 Department of Cardiac Surgery, Bieganski Hospital, Łódź, Poland; 42 Pediatric Cardiac Surgery, Jagiellonian University, Krakow, Poland; 43 Department of Cardiac Surgery, 4Th Military Hospital, Wrocław, Poland; 44 Subcarpathian Center for Cardiovascular Intervention, Sanok, Poland; 45 Department of Paediatric Cardiac Surgery, Poznan University of Medical Sciences, Poznań, Poland; 46 2nd Department of Cardiac Surgery, Medical University of Silesia, Katowice, Poland; 47 Division of Cardiac Surgery, Heart and Lung Transplantation and Mechanical Circulatory Support, Silesian Center for Heart Disease, Zabrze, Poland; 48 Department of Cardiac Surgery, Voivodeship Specialist Hospital of Olsztyn, Olsztyn, Poland

**Keywords:** Interventional cardiology, Risk factors

## Abstract

Surgical intervention in the setting of cardiogenic shock (CS) is burdened with high mortality. Due to acute condition, detailed diagnoses and risk assessment is often precluded. Atrial fibrillation (AF) is a risk factor for perioperative complications and worse survival but little is known about AF patients operated in CS. Current analysis aimed to determine prognostic impact of preoperative AF in patients undergoing heart surgery in CS. We analyzed data from the Polish National Registry of Cardiac Surgery (KROK) Procedures. Between 2012 and 2021, 332,109 patients underwent cardiac surgery in 37 centers; 4852 (1.5%) patients presented with CS. Of those 624 (13%) patients had AF history. Cox proportional hazards models were used for computations. Propensity score (nearest neighbor) matching for the comparison of patients with and without AF was performed. Median follow-up was 4.6 years (max.10.0), mean age was 62 (± 15) years and 68% patients were men. Thirty-day mortality was 36% (1728 patients). The origin of CS included acute myocardial infarction (1751 patients, 36%), acute aortic dissection (1075 patients, 22%) and valvular dysfunction (610 patients, 13%). In an unadjusted analysis, patients with underlying AF had almost 20% higher mortality risk (HR 1.19, 95% CIs 1.06–1.34; *P* = 0.004). Propensity score matching returned 597 pairs with similar baseline characteristics; AF remained a significant prognostic factor for worse survival (HR 1.19, 95% CI 1.00–1.40; *P* = 0.045). Among patients with CS referred for cardiac surgery, history of AF was a significant risk factor for mortality. Role of concomitant AF ablation and/or left atrial appendage occlusion or more aggressive perioperative circulatory support should be addressed in the future.

## Introduction

Cardiogenic shock (CS)-related condition at the time of cardiac surgery procedures is a common cause of mortality and its management remains a major challenge despite advances in therapeutic options including mechanical cardiovascular support (MCS)^1–3^. Some of the reversible causes of cardiogenic shock can be successfully managed surgically, provided they are diagnosed quickly before damage to the myocardium is permanent and recovery unlikely^4–6^. Regardless, cardiac surgery in patients with cardiogenic shock is often burdened with excessive risk^7–9^, for the following reasons: (1) detailed diagnostic process might have been not performed due to extremely compromised patient hemodynamic condition; (2) surgery tends to focus on the main objective which is to reverse the CS with borderline coronary lesions or moderate valve insufficiencies seldom addressed; (3) risk of postoperative complications is much higher due to end-organ hypoperfusion and dysfunction at baseline; and finally; (4) post-cardiotomy shock from low cardiac output syndrome (LCOS) is more likely to develop in these patients and postoperative MCS, such as veno-arterial extracorporeal membrane oxygenation (V-A ECMO) and other type of temporary or durable ventricle assist device (VAD), may be necessary alongside pharmacological support to stabilize the patients in this critical condition^10^.

Atrial fibrillation (AF) is the most common arrhythmia worldwide and its prevalence is higher in patients with coronary artery—(CAD) or valve disease^11^. The effect of untreated AF on long-term prognosis, both in patients who need cardiac surgery and in patients who do not, is well known^12–14^. On the other hand, the available evidence on whether and how pre-existing AF is complicating cardiogenic shock is limited to acute myocardial infarction (AMI) induced CS (AMI-CS)^15–17^ but poorly investigated in surgically treated CS patients. This is the first report to address early complications and long-term survival in patients undergoing heart surgery for CS with respect to pre-existing atrial fibrillation.

## Methods

Data were collected in a retrospective fashion from the KROK (Polish National Registry of Cardiac Surgery Procedures) registry (available at: www.krok.csioz.gov.pl). The registry is an ongoing, nationwide, multi-institutional registry of heart surgery procedures in Poland; the details on registry conception and design were described previously^18–20^. Centers enrolling patients in the KROK registry are required to transfer the data concerning every cardiac surgery to the central database in the National Centre for Healthcare Information Systems at the Ministry of Health and are financially liable for data integrity and completeness. Follow-up data regarding mortality were obtained from the National Health Fund—the nationwide, obligatory, public health insurance institution in Poland and incorporated to the registry. The study was approved by the Institutional Board of Central Clinical Hospital of the Ministry of Interior, Centre of Postgraduate Medical Education, Warsaw, Poland and adheres to Helsinki Declaration as revised in 2013. Due to the anonymization of registry data, patient informed consent was waived by the Institutional Board of Central Clinical Hospital of the Ministry of Interior, Centre of Postgraduate Medical Education, Warsaw, Poland.

### Study population

**Figure 1:**
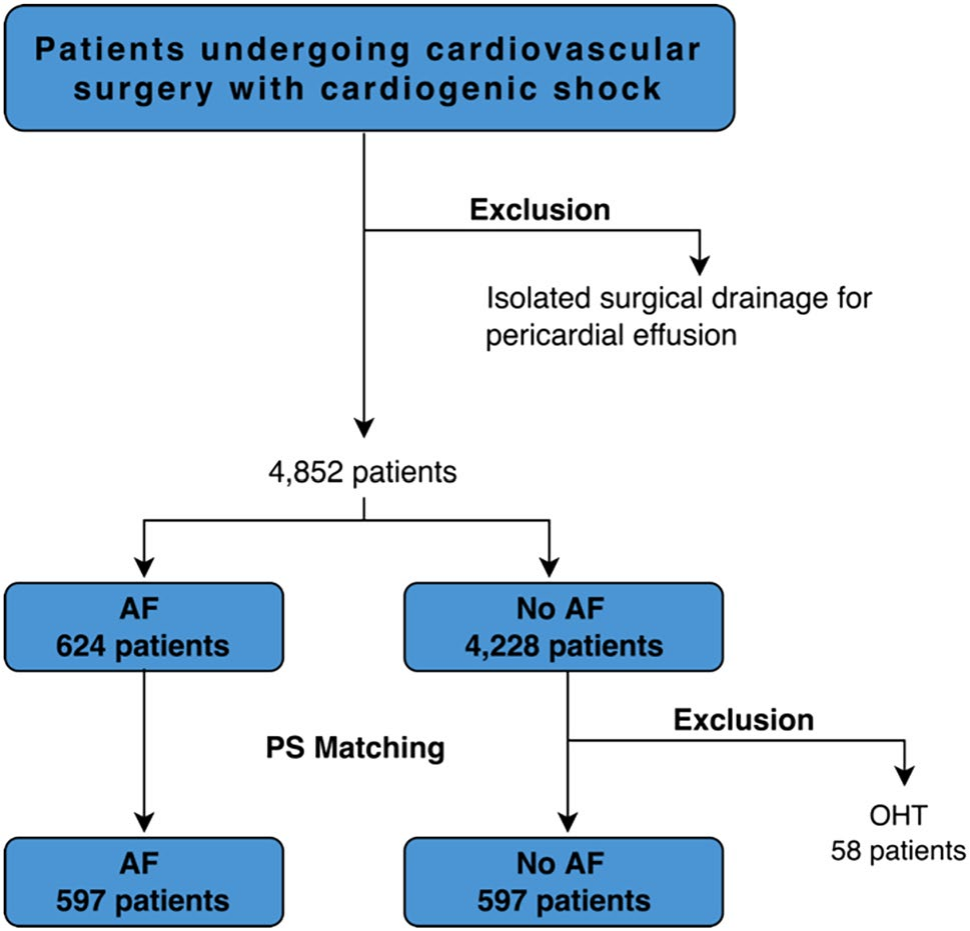
Study flow-chart. AF, atrial fibrillation; PS, propensity score; OHT, orthotopic heart transplantation.

The registry included all adult patients undergoing heart surgery between and 1st Jan 2012 and 31st Dec 2021 and presenting with cardiogenic shock due to all causes. Only patients undergoing heart surgery for isolated pericardial effusion were excluded. Cardiogenic shock in the KROK registry was defined as per SHOCK trial criteria^21^ until 2016; from then on, European Society of Cardiology Heart Failure guidelines^22^ criteria were imposed (both available in the Supplementary Methods). Diagnosis of cardiogenic shock was left to discretion of treating physician. We divided the study cohort into patients with documented history of AF (prior-to-admission ICD-9/ICD-10 documentation codes, or on-admission ECG) before the index surgery, and patients without documentation of AF. Post-operative AF was not recorded and therefore not considered. The study flow chart of the present analysis is shown in Fig. 1.

### Clinical variables and endpoints

For patients undergoing heart surgery, we considered and reported 3 categories of variables: (1) baseline demographics: age, gender, EuroSCORE II^23^ and its single components; (2) extent of coronary artery disease (CAD) and/or valvular and/or aortic disease and (3) surgical variables: urgency, operative technique (e.g. on-pump vs. off-pump coronary artery bypass grafting [CABG] surgery). The primary endpoint was death from any cause reported at 30 days and longest available follow-up for the comparison of AF and non-AF patients. In-hospital outcomes and lengths of stays in the intensive care unit (ICU) and hospital (HLoS) are reported and compared as well. Baseline clinical-, procedural- and outcome data at follow-up were entered into prespecified electronic case report forms. Follow-up status with respect to all-cause mortality is validated by Polish National Health Fund and incorporated into the KROK registry.

### Statistical analysis

Registry records with > 5% of missing data were not considered; in those with < 5%, missing data were input by artificial neural networks^24^. Continuous variables were summarized as mean ± standard deviation if normally distributed; non-normal distributions were summarized as median and interquartile range (IQR) and compared with the Mann–Whitney U test or standard t test as appropriate. Categorical variables (number [%]) were compared with the Fisher’s exact test. Risk ratios (RRs) were used primarily for 30-day/in-hospital outcomes. Univariable and multivariable analyses to determine predictors of mortality were conducted. Similarly, we carried out univariable and multivariable analyses to identify the factors associated with the prevalence of AF. We built a non-parsimonious model including variables identified in multivariable analyses^25^ for propensity score matching (PSM); a 1 to 1 nearest neighbor matching was performed with replacement (caliper 0.2); the overall long-term mortality was assessed with Kaplan–Meier curves fitted before (unadjusted model) and after propensity score matching. Inverse probability weighting (IPW) was performed as sensitivity analysis in order not to exclude from adjusted analysis potentially substantial proportion of participants. Cox regression was used to determine long-term hazard ratio (HR) for all-cause mortality as stratified by AF and non-AF patients. As a further sensitivity analysis to assess the survival in AF and non-AF subsets, we further stratified patients according to pre-defined subgroups. STATA MP v13.0 software (StataCorp, College Station, TX USA) and the packages “psmatch2”, “robust”, “optmatch”, “matchIt” and “CRTgeeDR" in R Core Team 2013 were used.

## Results

### Baseline demographics

Within the investigated time-frames, 332,109 patients underwent cardiac surgery; Of those 4852 (1.5%) patients presented with CS and their registry records provided data relevant for the analyses. Preoperative AF was documented in 624 of 4852 (12.8%) patients, the mean age was 62 years and 3297 (68%) patients were men. Baseline characteristics of unadjusted group of patients are further available as Supplementary Table 1. Presence of underlying atrial fibrillation was associated with age (*P* < 0.001), repeat surgery (*P* < 0.001); diabetes (*P* < 0.001); hypertension (*P* = 0.002); chronic kidney and pulmonary disease (*P* = 0.026 and 0.005 respectively) as well as mitral valve disease (*P* < 0.001); patients presenting with coronary disease (*P* = 0.005) and acute aortic dissection (*P* = 0.021) less frequently had underlying AF in multivariable analysis (Supplementary Table 2). The origin of CS included acute myocardial infarction (36%), acute aortic dissection (22%), and valvular dysfunction (13%). Other etiologies of CS are shown in Supplementary Fig. 1 and Supplementary Table 3. Acute MI mechanical complications (free wall rupture, papillary muscle rupture, ventricular septal defect and left ventricle aneurysm) constituted 334 (6.9%) of cardiogenic shock causes (Supplementary Fig. 1).

Mechanical circulatory support was used preoperatively in 920 (21.4%) patients, and most commonly included intra-aortic balloon pump in 876 (18.1%) patients; followed by ECMO in 78 (3.0%) patients and VAD in 97 (2.0%).

Coronary artery bypass grafting was most commonly performed procedure [1594 pts (32.9%)] followed by aortic dissection repair in 957 (19.7%); aortic, mitral and tricuspid valve repair or replacement surgery was performed in 727 (15.0%), 551 (11.4%) and 140 (2.9%) cases respectively. Fifty-eight (1.2%) patients underwent orthotopic heart transplantation while 101 (2.1%) underwent VAD implantation. Median ICU length of stay was 101.5 h [Interquartile range (IQR) 47.3–213.6] and HLoS among those who survived to discharge 9.2 days (IQR 5.7–16.6). Surgical data are reported in Supplementary Table 4.

Thirty-day mortality was 35.6%. In-hospital complications are available as Supplementary Table 5. In multivariable analysis, age (*P* < 0.001); repeat surgery (*P* = 0.012); hypertension (*P* = 0.001); chronic kidney disease (*P* < 0.001); peripheral artery disease (*P* < 0.001); mechanical ventilation (*P* < 0.001) and surgical urgency (*P* < 0.001) were associated with long-term mortality (Supplementary Table 6). In an unadjusted analysis, patients with AF had almost 20% higher mortality risk (HR 1.19, 95% CIs 1.06–1.34; *P* = 0.004) (Supplementary Fig. 2).

### PS-matched analysis

We performed a propensity score analysis after the exclusion of orthotopic heart transplantation patients. After the PS-matching 597 pairs were identified (Fig. 1). Baseline characteristics of the study cohort are summarized in Table 1. Patients with AF had more previous percutaneous coronary artery intervention (13.7% vs. 18.9%; *P* = 0.01), whereas no other significant differences regarding the prevalence of cardiovascular risk factors and comorbidities were seen (Table 1, Supplementary Fig. 3—SMD figure Love plot, Supplementary Fig. 4—PS distribution plot). Principal causes of cardiogenic shock are listed in Supplementary Table 7. We observed no marked differences between AF and no AF patients in terms of CS origin. Around 30% of patients in both groups were operated on shortly after MI (6.5% had mechanical AMI complications). In 15% of patients in both groups acute aortic dissection was the indication for emergent surgery, while pulmonary embolism and infective endocarditis accounted for around 10% in each group.

**Table 1:** Preoperative characteristics after PS-matching.

Variable	PS-matched patients		
	No AF (597)	AF (597)	*P*_value_
Baseline characteristics			
Age (years)	68 [60–76]	67 [60–75]	0.410
Female gender	214 (35.8)	217 (36.3)	0.904
EUROSCORE II (median [IQR])	20.37 [8.47–39.98]	23.06 [10.32–41.18]	0.096
Diabetes	214 (35.8)	231 (38.7)	0.338
Smoking	324 (54.3)	356 (59.6)	0.070
Hypertension	471 (78.9)	465 (77.9)	0.725
Hyperlipidemia	293 (49.1)	306 (51.3)	0.487
BMI (median [IQR])	27.3 [24.4–30.5]	27.5 [24.6–30.9]	0.474
Renal impairment	276 (46.2)	283 (47.4)	0.728
Dialysis	30 (5.0)	31 (5.2)	1.000
Pulmonary hypertension	222 (37.2)	224 (37.5)	0.952
PVD	207 (34.7)	208 (34.8)	1.000
Carotid disease	51 (8.5)	68 (11.4)	0.122
Previous Stroke	38 (6.4)	54 (9.0)	0.103
Asthma/COPD	113 (18.9)	121 (20.3)	0.610
LVEF	40 [30–50]	40 [30–50]	0.651
CAD	189 (31.7)	189 (31.7)	1.000
LM disease	50 (8.4)	50 (8.4)	1.000
Previous MI	174 (29.1)	198 (33.2)	0.151
Previous PCI	82 (13.7)	113 (18.9)	0.019

PS, propensity score; IQR, interquartile range; BMI, body mass index; PVD, Peripheral Vascular Disease; COPD, chronic obstructive lung disease; LVEF, left ventricle ejection fraction; CAD, coronary artery disease; LM, left main; MI, myocardial infarction; PCI, percutaneous intervention.

Surgical data are listed in Table 2. There was a trend towards higher prevelance of hemodynamic instability, defined as the use of iv inotropes (65.3% vs. 70.4%; *P* = 0.072) in the AF group. Coronary artery bypass grafting (23.5%) and mitral valve procedure (21.6%) were the most commonly performed procedures without significant differences between AF and no-AF patients. In the AF group, the tricuspid valve procedures (4% vs. 7.2%; *P* = 0.023) and surgical pulmonary embolectomy rates (1% vs. 3%; *P* = 0.021) were higher. Among patients with AF concomitant cardiac ablation was performed in 6 patients (1%) and left atrial appendage (LAA) closure in 12 (2%).

**Table 2:** Operative characteristics after PS-matching.

Variable	PS-matched patients		
	No AF (597)	AF (597)	*P*_value_
Procedural characteristics			
iv. inotropes	390 (65.3)	420 (70.4)	0.072
iv. nitrates	228 (38.2)	231 (38.7)	0.905
IABP	96 (16.1)	95 (15.9)	1.000
VAD pre-op	12 (2.0)	12 (2.0)	1.000
ECMO	10 (1.7)	19 (3.2)	0.131
Mechanical ventilation	223 (37.4)	225 (37.7)	0.952
Urgency			
Urgent	158 (26.5)	185 (31.0)	0.096
Emergent	287 (48.1)	273 (45.7)	0.451
Salvage	152 (25.5)	139 (23.3)	0.419
Surgery			
CPB time* (median [IQR])	131 [95–189]	131 [95–182]	0.712
X-clamp time* (median [IQR])	80 [51–106]	79 [53–112]	0.726
Redo surgery	63 (10.6)	103 (17.3)	0.001
MVR	123 (20.6)	135 (22.6)	0.439
AVR	106 (17.8)	97 (16.2)	0.538
TVPR	24 (4.0)	43 (7.2)	0.023
CABG	150 (25.1)	134 (22.4)	0.308
Post inf VSD	15 (2.5)	26 (4.4)	0.111
Aorta repair	110 (18.4)	101 (16.9)	0.544
ECMO (central cannulation)	30 (5.0)	36 (5.7)	0.527
VAD implantation	7 (1.2)	21 (3.5)	0.012
Trauma	29 (4.9)	30 (5.0)	1.000
Tumor	2 (0.3)	2 (0.3)	1.000
Embolectomy	6 (1.0)	18 (3.0)	0.021
Other	107 (17.9)	129 (21.6)	0.127

PS, propensity score; IABP, intra-aortic balloon pump; iv., intravenous; OPCAB, Off-Pump Coronary Artery Bypass; CPB, cardiopulmonary bypass; LAAO, left atrial appendage occlusion; VSD, ventricular septal defect; SD, Standard Deviation.

*Missing data.

The use of mechanical circulatory support (pre-operative ventricular assist device (2% vs. 2%; *P* = 1.000) and extracorporeal membrane oxygenation (1.7% vs. 3.2%; *P* = 0.131) was similar in both groups.

**Figure 2:**
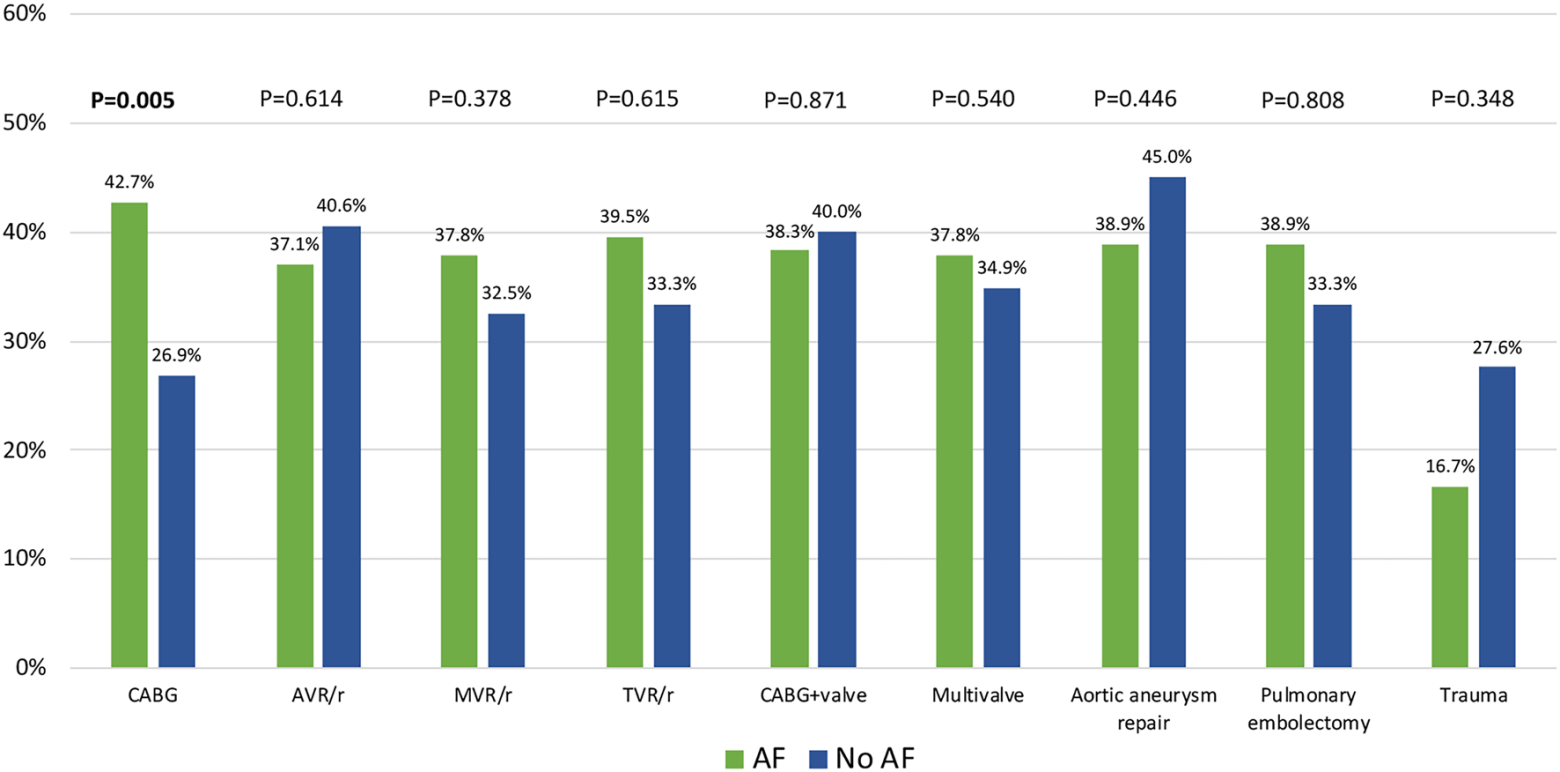
30-day mortality in AF and no AF groups according to the type of surgery. CABG, Coronary Artery Bypass Grafting; AVR, aortic valve replacement; MVR, mitral valve replacement; TVR/r, tricuspid valve replacement/repair.

**Figure 3:**
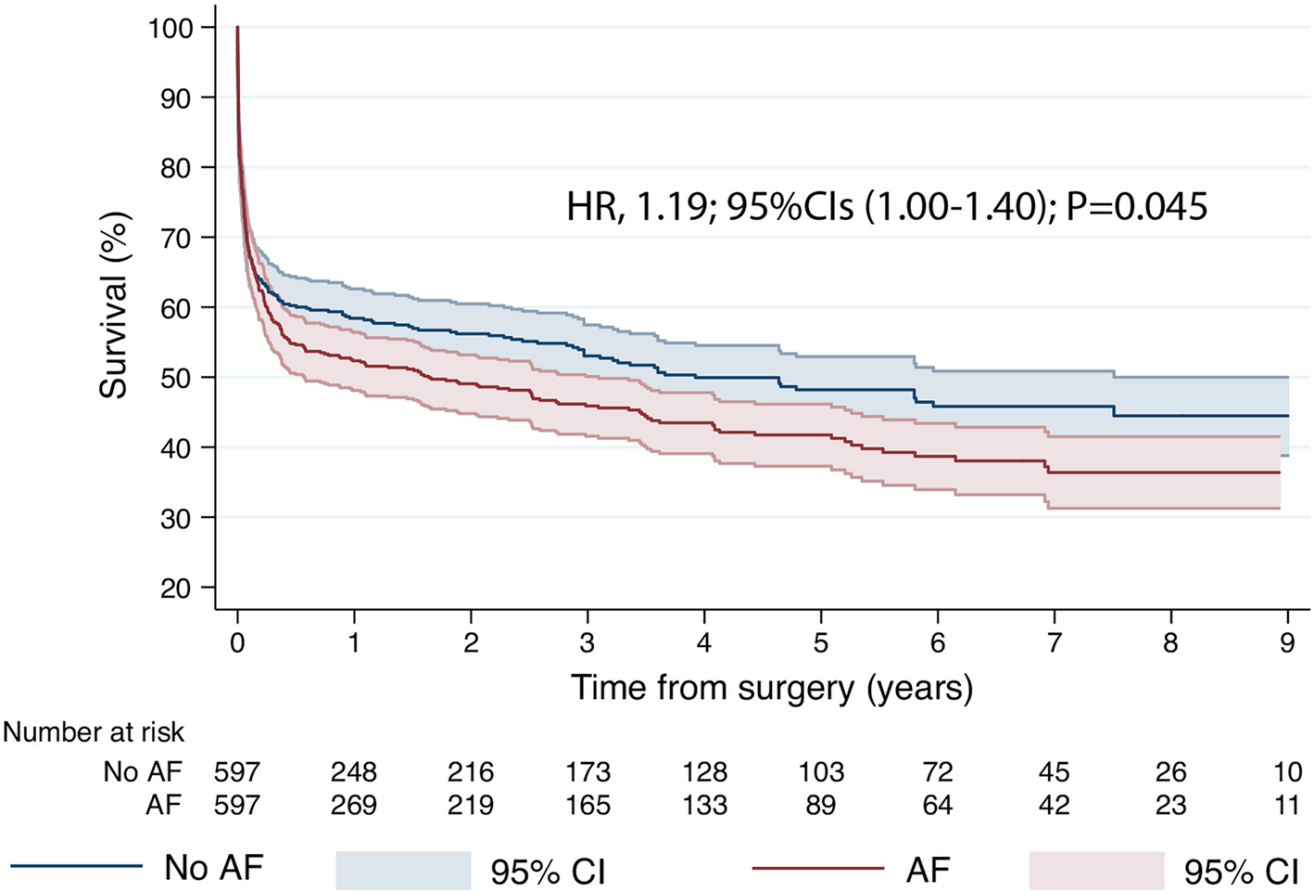
Adjusted Kaplan–Meier curve displaying survival according to presence or absence of AF. AF, atrial fibrillation; HR, hazard ratio; CI, Confidence Intervals.

In hospital outcomes are reported in Supplementary Table 8. The major postoperative outcomes: severe bleeding requiring re-thoracotomy, respiratory failure, neurological and gastrointestinal complications; superficial and deep sternal wound infection and the use of ECMO and intra-aortic balloon pump was similar in both groups. In the PS-matched analysis, total 30-day mortality was 33.6% and was numerically higher in AF group (34.7 vs. 32.5%; *P* = 0.462) with incidence rates varying across type of surgical procedures; AAD repair had highest (41.8%), followed by AVR/r (38.9%), CABG + valve (38.6%), TVR/r 36.8%, multivalve surgery 36.2% and mitral valve procedures (35.3%), without significant differences between AF and No AF groups but CABG group (42.7 vs. 26.9%; *P* = 0.005) in favor of no AF (Fig. 2). Median follow-up was 4.6 years (max.10.0 years) and it was 100% complete for the mortality outcome; AF remained associated with worse survival (HR 1.19, 95% CI 1.00–1.40; *P* = 0.045) (Fig. 3) at long term.

**Figure 4:**
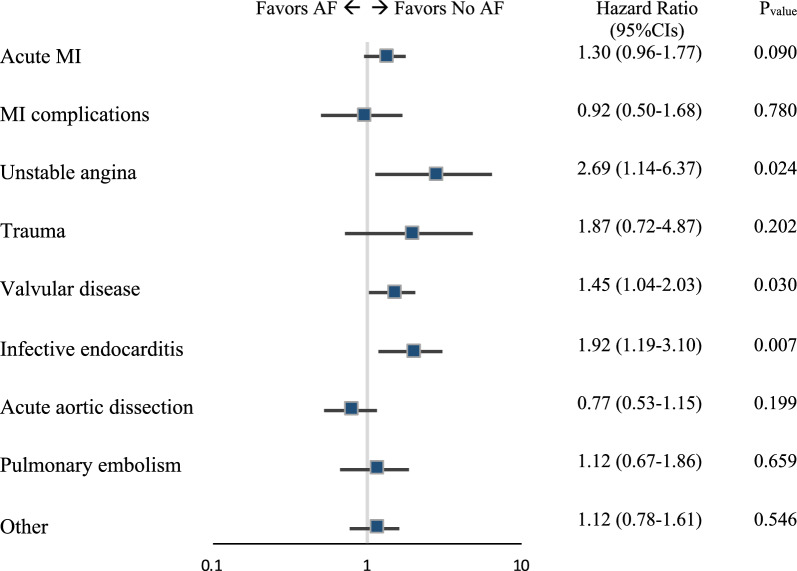
The influence of preoperative AF on mortality according to CS origin. AF, atrial fibrillation; CI, confidence intervals; MI, myocardial infarction.

Proportional hazard assumption was not violated (*P* = 0.439) as also graphically assessed (Supplementary Fig. 5 and 6). In the IPTW analysis, AF was still associated with worse long-term survival with mortality increased in the AF cohort by 3.51% each year (95% CI 0.03–6.74%, *P* = 0.033). In the subgroup analysis, the harmful effect of AF on long-term mortality was seen in patients initially presenting with unstable coronary artery disease (*P* = 0.024) and valvular disease (*P* = 0.030), in particular IE (*P* = 0.007) (Fig. 4).

## Discussion

To the best of our knowledge, this is the first study from a large national inpatient database to analyze the prognostic impact of underlying AF in various setting of CS requiring heart surgery. As major findings, history of AF strongly impacts (1) the survival at 30 days driven by reduction of mortality in patients undergoing CABG surgery and (2) and is associated with higher long-term mortality regardless of the etiology of CS. (3) postoperative complications were similar in patients with and without documented AF during index hospital stay; furthermore, (4) concomitant ablation of AF and closure of left atrial appendage are rarely performed during cardiac procedure for CS.

Atrial fibrillation is the most common cardiac arrhythmia in the general population and a lifetime risk of > 20% after the age of 55^11,26^. Its prevalence is estimated to at least double with ageing population^11^. Stroke is the most feared complication in patients with AF, however it also impacts on clinical outcome in specific clinical conditions such as AMI and heart failure or following cardiac surgery procedure^13–17,27^. As many as 28% of the patients admitted for heart surgery procedure present with AF with increasing rates depending on the presence of valvular dysfunction and extent of the cardiac disease^28^. AF is a well-known marker of high-risk patients and a predictor of postoperative complications including mortality, postoperative stroke, renal failure, prolonged ventilation, reoperation, and deep sternal wound infection^13^. Patients with preoperative diagnosis of AF also experience a higher adjusted long-term risk of all-cause death and of a cumulative risk of stroke and systemic embolism compared to those without^13^.

### AF and CS post AMI

The prognostic impact of AF in the setting of CS complicating AMI has mostly been reported after percutaneous procedures. From the IABP-SHOCK II trial (600 patients enrolled, 169 with AF versus 431 without), there were no significant differences with respect to mortality at 30 days and 12 months between patients with and without AF^29^. Similarly, the rates of recurrent MI, repeat revascularization, and stroke did not differ between groups. The authors did not observe any interaction between the impact of IABP on clinical outcome and the prevalence of AF. Reflecting the above were the findings reported in a sub-analysis of the Culprit Lesion Only PCI versus Multivessel PCI in Cardiogenic Shock trial^15^. The study included 686 patients (142 with AF history on admission, or newly detected AF during index hospitalization) and AF was not a significant predictor of 30-day and 1-year all-cause mortality. However, patients with AF already on admission (90 of 142), showed higher all-cause mortality at 30 days (58% vs. 37%; *P* = 0.02) and 1 year (63% vs. 39%; *P* = 0.004) compared with patients with newly detected AF during hospital stay. Furthermore, AF was associated with longer time to hemodynamic stabilization (4 vs. 3 days; *P* = 0.04) at 30 days. In another PS-matched study from NIS registry including 840 patients (420 with AF) who underwent PCI while on percutaneous VAD (Impella®) because of CS complicating AMI, all-cause in-hospital mortality rates between the two groups were similar (40.5% vs. 36.7%, *P* = 0.245). However, the AF group experienced a significantly higher rate of postprocedural respiratory complications (9.5% vs. 4.8%; *P* = 0.007), fewer routine discharges (13.1% vs. 30.2%; *P* < 0.001) and more frequent transfers to other healthcare facilities (27.3% vs. 17.8%; *P* < 0.001). The mean LOS (12 vs. 9 days; *P* < 0.001) and hospital charges ($308,478 vs. $277,982; *P* = 0.008 ) were higher in the AF group^16^.

### AF and CS—surgical strategy

The impact of preoperative AF in patients requiring heart surgery for CS is poorly investigated and reported. In our study, CAD remains the major cause of CS and CABG remains the most common surgical treatment, respectively. The current guidelines do not exclude a role for emergency CABG that is usually regarded as the last resource and only in a very limited percentage of patients^30^. Patients undergoing isolated CABG for CS suffer up to 20% higher mortality rates comparing to those without and this occurs also with milder degrees of CS^31,32^. In one recent analysis from the STS database, of the 5259 patients with AMI complicated by CS who underwent CABG during the study period, 665 (12.6%) patients had AF which in a multivariable logistic regression analysis was associated with increased operative mortality (HR 1.44, 95% CI [1.18–1.77]; *P* < 0.001)^33^.

Recent reports on surgery for mitral papillary muscle rupture and CS from the Japan cardiovascular surgery database (196 patients, 140 CS) and STS database (1342 patients, 759 CS) do not address this issue (the former) or describe no impact of preoperative arrhythmias as predictors of operative mortality in multivariable logistic regression model (the latter)^34,35^. Sagakuchi et al. identified 1397 patients undergoing surgical repair of post-MI VSD (61.5% CS) from the national Japanese database and concluded that preoperative AF was not a significant prognostic factor (HR 0.79, 95% CI [0.50–1.23]; *P* = 0.29 in the multivariable analysis)^36^. Similarly, no relationship was observed between the prevalence of the AF and survival in the UK National Adult Cardiac Surgery Audit of post‐infarct ventricular septal defect repair (5.0% among survivors, 5.9% among non-survivors; *P* = 0.6)^37^. Accordingly, in our study, we did not observe differences in survival in the MI mechanical complications subgroup. However, we noted a significant relationship among patients with different CS etiology, particularly CAD and valvular decease.

One interesting finding of the current analysis is the low utilization rate of MCS devices in patients with CS in anticipation of or following the surgical treatment. In the setting of CS, temporary MCS can help to stabilize patients and grant time for decision-making about the definitive management^31^. In a recent STS report, AF occurrence in patients with AMI and CS undergoing CABG was higher in the MCS group suggesting a further negative hemodynamic impact of this arrhythmia^31^. In our analysis only 19.0% patients received MCS, and these most commonly included IABP (18.1%); followed by ECMO in 78 (3.0%) patients and VAD in 97 (2.0%). What is reflected in the present analysis is the approach to rush the patient to the OR and stabilize the condition with CPB in most cases rather than stabilize the patient first in the ICU.

### “Anti-AF” approaches

This study shows that ablation of AF or LAAO during heart surgery for patients in CS is very seldom performed. From the 2020 STS report, only 18 patients among 1342 (1.3%) that underwent mitral valve surgery for ischemic papillary muscle rupture received ablation and in three major randomized trial on LAA closure (LAOS I–III) non-elective surgical cases were excluded by the study design^38–40^. Conditions related to CS requiring surgery are demanding and challenging operations and it is perfectly understandable that management of the cause of CS should be the priority. The current analysis could not address AF surgical management; yet, because of lower mortality in the no-AF matched, it may suggest there is a potential to reduce both early and long-term mortality when AF is addressed as well. Indeed, previous observational studies suggested similarly lower risk of long-term mortality in patients undergoing surgical ablation concomitant to CABG w/wo valvular procedure in patients in critical condition, with pre-op IABP and on pharmacological inotropic support^20^.

### Limitations

There are certain limitations to the current retrospective study that need to be acknowledged; firstly, the registry did not collect, at the time of conception, the data regarding long-term outcomes other than all-cause mortality e.g. long-term stroke, rehospitalization for heart failure, repeat revascularization, re-do surgery and other procedures e.g. catheter ablation or PCI; these could further enhance the registry and might have influenced the remote outcome as well. Secondly, the registry does not collect the data regarding medical therapy especially regarding oral anticoagulation (OAC) therapy. Information regarding OAC in both pre-existing AF and postoperative AF, should one occur, would shed light to evolving thromboembolic risk in shock patients given the lack of unanimous recommendations regarding OAC institution in POAF and OAC postoperative reinstitution in pre-existing AF. Thirdly, certain detailed baseline and operative data such as AF type and duration were not collected by the registry; information on the timing of interventions, delay to surgery, duration of pre-op IABP, doses of inotropes and certain characteristics of mechanical ventilation and other ICU variables are missing. Finally, while PSM accounted for the variables included in the EuroSCORE II and other surgically relevant characteristics minimizing selection bias in an attempt to even baseline patients’ characteristics, unmeasured biases and confounders may remain, in particular in the setting of cardiogenic shock, making the association between AF and higher mortality in cardiogenic shock valid only to the extent an analysis of a non-RCTs study allows. On the other hand, multivariable analyses fully support the concept of AF as a hallmark of worse baseline condition and higher risk independently associated with worse prognosis both at early and long-term follow-up. The optimal timing of surgical intervention in patients with CS that could benefit of preoperative MCS is a matter of further debate not addressed by this study.

## Conclusions

Among patients with CS referred for cardiac surgery, history of AF was a significant risk factor for long-term mortality. Thirty-day mortality was 36% with a significant difference between AF and no-AF in favour of the latter in subgroup of patients undergoing CABG. Addressing AF by concomitant ablation and/or left atrial appendage closure at the time of surgery may be considered to reduce thromboembolic risk and worsening of heart failure even in these highest risk patients. However, additional and dedicated studies investigating patients in CS and affected by preoperative AF should be undertaken to carefully analyze the actual impact and related therapeutic treatment to abolish such a cardiac arrhythmia in this peculiar hemodynamic setting.

### Supplementary Information

Supplementary Information.

## Data Availability

The datasets generated during and/or analyzed during the current study are available from the corresponding author on reasonable request.
