# Supplementary material for: Prognostic impact of preoperative atrial fibrillation in patients undergoing heart surgery in cardiogenic shock
Source: Sci Rep. 2023 Dec 9;13:21818. doi: 10.1038/s41598-023-47642-3 (PMC10710503; doi:10.1038/s41598-023-47642-3)
Supplement: Supplementary file 1 — Supplementary Information. [file 41598_2023_47642_MOESM1_ESM.docx]

**KROK Investigators:**

Lech Anisimowicz MD, PhD ^1^; Andrzej Biederman MD, PhD ^2^; Dariusz Borkowski MD ^3^; Mirosław Brykczyński MD, PhD ^4^; Paweł Bugajski MD, PhD ^5^; Marian Burysz ^6^, Paweł Cholewiński ^3^; Romuald Cichoń MD, PhD ^7,8^; Marek Cisowski MD, PhD ^9^; Antoni Dziatkowiak MD, PhD ^10^; Tadeusz Gburek MD ^11^; Witold Gerber MD ^9^; Leszek Gryszko MD ^12^; Ireneusz Haponiuk MD ^13^; Piotr Hendzel MD, PhD ^14^; Stanisław Jabłonka MD, PhD ^15^; Krzysztof Jarmoszewicz MD ^16^; Ryszard Jaszewski MD, PhD ^18^; Marek Jemielity MD, PhD ^19^; Ryszard Kalawski MD, PhD ^5^; Bogusław Kapelak MD, PhD ^10^; Maciej A. Karolczak MD, PhD ^20^; Jacek Kaperczak MD ^21^; Piotr Knapik MD, PhD ^22^; Michał Krejca MD, PhD ^18^; Wojciech Kustrzycki MD, PhD ^23^; Mariusz Kuśmierczyk MD, PhD ^8^; Paweł Kwinecki MD ^7^; Leszek Markuszewski MD, PhD ^25^; Maurycy Missima MD ^26^; Jacek J Moll MD, PhD ^27^; Wojciech Ogorzeja MD ^6^; Jacek Pająk MD ^20^; Michał Pasierski ^28^;Wojciech Pawliszak MD ^1^; Edward Pietrzyk MD ^29^; Grzegorz Religa MD ^30^; Jan Rogowski MD, PhD ^31^; Jacek Różański MD, PhD ^24^; Jerzy Sadowski MD, PhD ^10^; Girish Sharma MD ^7^; Janusz Skalski MD, PhD ^32^; Jacek Skiba MD ^33^; Ryszard Stanisławski MD ^17^; Janusz Stążka MD, PhD ^15^; Sebastian Stec MD, PhD ^34^; Piotr Stępiński MD ^17^; Grzegorz Suwalski MD ^12^; Kazimierz Suwalski MD, PhD ^12^; Łukasz Tułecki MD ^11^; Waldemar Wierzba MD ^12^; Michał Wojtalik MD, PhD ^35^; Stanisław Woś MD, PhD ^36^; Michał Oskar Zembala MD, PhD ^37^ and Piotr Żelazny MD ^38^.

^1^ Department of Cardiac Surgery, University Hospital, Bydgoszcz, Poland

^2^ Cardiac Surgery Department, Medicover Hospital, Warsaw, Poland

^3^ Department of Cardiac Surgery Masovian Specialistic Hospital of Radom, Radom, Poland

^4^ Department of Cardiac Surgery, Pomeranian Medical University, Szczecin, Poland

^5^ Department of Cardiosurgery, J. Struś Hospital, Poznań, Poland

^6^ Department of Cardiac Surgery, Regional Specialist Hospital, Grudziadz, Poland

^7^ Department of Cardiac Surgery, Medinet Heart Center Ltd, Wroclaw, Poland

^8^ Department of Cardiovascular Surgery, University Clinical Center of the Medical University of Warsaw, Poland

^9^ Department of Cardiac Surgery, American Heart of Poland, Bielsko-Biała, Poland

^10^ Department of Cardiovascular Surgery and Transplantology, Jagiellonian University Medical College, John Paul II Hospital, Krakow, Poland

^11^ Department of Cardiac Surgery, The Pope John Paul II Province Hospital, Zamość, Poland

^12^ Department of Cardiac Surgery, Military Institute of Medicine, Warsaw, Poland

^13^ Department of Pediatric Cardiac Surgery, Pomeranian Traumatology Center, Gdańsk, Poland

^14^ Medical University of Warsaw, Warsaw, Poland.

^15^ Cardiac Surgery Department, Medical University of Lublin, Lublin, Poland

^16^ Department of Cardiac Surgery, Ceynowa Specialist Hospital in Wejherowo, Poland

^17^ Lower Silesian Center for Heart Diseases, Nowa Sól, Poland

^18^ Department of Cardiac Surgery, Medical University of Lodz, Lodz, Poland

^19^ Department of Cardiac Surgery and Transplantology, Poznan University of Medical Sciences

^20^ Department of Cardiac and General Pediatric Surgery, Medical University of Warsaw, Warszawa, Poland

^21^ Department of Cardiac Surgery, University Hospital, Institute of Medical Sciences, University of Opole, Opole, Poland

^22^ Department of Anesthesiology and Intensive Therapy, Silesian Centre for Heart Diseases in Zabrze, Medical University of Silesia, Poland

^23^ Department of Cardiac Surgery, Wroclaw Medical University, Wroclaw, Poland

^24^ Department of Cardiac Surgery and Transplantology, National Institute of Cardiology, Warszawa, Poland

^25^ Department of Medicine, Faculty of Medical Sciences and Health Sciences, Kazimierz Pulaski University of Technology and Humanities in Radom, Radom, Poland

^26^ Cardiology and Cardiac Surgery Department, 11th Military Research Hospital and Polyclinic IPHC in Bydgoszcz, Bydgoszcz, Poland

^27^ Department of Cardiac Surgery, Polish Mother's Memorial Hospital Research Institute, Lodz, Poland

^28^ Clinical Department of Cardiac Surgery and Transplantology, National Medical Institute of the Ministry of Interior and Administration, Centre of Postgraduate Medical Education, Warsaw, Poland

^29^ Department of Cardiac Surgery, Swietokrzyskie Cardiology Center, Kielce, Poland

^30^ Department of Cardiac Surgery, Bieganski Hospital, Łódź, Poland

^31^ Department of Cardiac and Vascular Surgery, Medical University of Gdansk, Gdańsk, Poland

^32^ Pediatric Cardiac Surgery, Jagiellonian University, Krakow, Poland

^33^ Department of Cardiac Surgery, 4th Military Hospital, Wrocław, Poland

^34^ Subcarpathian Center for Cardiovascular Intervention, Sanok, Poland

^35^ Department of Paediatric Cardiac Surgery, Poznan University of Medical Sciences, Poznan, Poland

^36^ 2nd Department of Cardiac Surgery, Medical University of Silesia, Katowice, Poland

^37^ Division of Cardiac Surgery, Heart and Lung Transplantation and Mechanical Circulatory Support, Silesian Center for Heart Disease, Zabrze, Poland

^38^ Department of Cardiac Surgery, Voivodeship Specialist Hospital of Olsztyn, Olsztyn, Poland
